# Chemical Characterization and Allelopathic Effects of *Ambrosia psilostachya* (Asteraceae)

**DOI:** 10.1002/cbdv.202502443

**Published:** 2025-11-14

**Authors:** Hesan Saberi, Ali Reza Yousefi, Majid Pouryousef, Somayeh Tokasi, Jafar Asghari Birbaneh, Marcello Iriti

**Affiliations:** ^1^ Department of Plant Production & Genetics Faculty of Agriculture University of Zanjan Zanjan Iran; ^2^ Plant Protection Research Department Guilan Agricultural and Natural Resources Research and Education Center, Agricultural Research Education and Extension Organization Rasht Iran; ^3^ Department of Agronomy and Plant Breeding Faculty of Agricultural Sciences University of Guilan Rasht Iran; ^4^ Department of Biomedical Surgical and Dental Sciences University of Milan Milan Italy; ^5^ National Interuniversity Consortium of Materials Science and Technology (INSTM) Firenze Italy

**Keywords:** allelopathy, essential oil, invasive plants, alien species, weeds

## Abstract

*Ambrosia* spp. have been introduced and distributed across different continents as an invasive alien plant species. The present study aimed to identify the chemical components of essential oil isolated from different parts of *Ambrosia psilostachya* and evaluate the phytotoxic effect of its aqueous extracts on the growth parameters of *Phaseolus vulgaris* L. (bean) and *Arachis hypogaea* L. (peanut) under greenhouse conditions. The chemical components of the essential oil of *A. psilostachya* were analyzed by gas chromatography‐mass spectrometry. Total 44 compounds, including isoaromadendrene epoxide (17.38 %), citral (15.13 %), caryophyllene (7.23 %), borneol (8.76%), (+)‐2‐bornanone (6.45 %), eucalyptol (4.61 %), and 22 compounds including endo‐borneol (22.76%), (+)‐2‐bornanone (8.08%), caryophyllene oxide (7.77%), citral (14.65%), *cis*‐verbenol (4.43%), and eucalyptol (4.72%) as the major components were identified and isolated in the shoots and roots, respectively. To evaluate the allelopathic effect of *A. psilostachya* on bean and peanut seedlings, the aqueous extracts were prepared at four concentrations (25%, 50%, 75%, and 100%). The extracts at the concentrations of 50% or higher had a significant inhibitory effect on yield, dry weight, height, and LAI in both species. The inhibitory activity of *A. psilostachya* may be attributed to its high content of isoaromadendrene epoxide, citral, caryophyllene, and caryophyllene oxide. This study highlights that the allelopathy effects of *A. psilostachya* are due to the action of its compounds, which should be analyzed in future studies, and an accurate evaluation of the allelopathy effects of this invasive plant requires comprehensive, long‐term studies in the environment.

## Graphical abstract

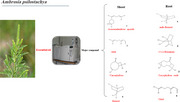

## 1 Introduction

Invasive plants seriously threaten flora by competing or allelopathy into the environment [1]. Allelopathy is a phenomenon in which living or dead plants produce and release chemical compounds [2] that can affect the germination or growth of other species can alter their performance, and ultimately plant community structure. Indeed, allelopathy is a mechanism to facilitate the spread of invasive species by releasing allelochemicals such as alkaloids, flavonoids, terpenoids, phenolics, etc. [3, 4]. Allelochemicals are released into the environment through root exudation, leaching, and decomposition from living or dead plants. The *Ambrosia* spp. represent one of the most invasive groups of weeds in the world, the invasion of them into field crops has caused concerns in many countries [5]. The effect of the allelopathic activities of *Ambrosia* spp. is documented in many literature [6, 7, 8]. According to [9] results, all extracts of *Ambrosia artemisiifolia* significantly reduced the germination of the *Zea mays* seeds. Similar findings have also been reported by [10] that *A. artemisiifolia* inhibits the early growth of tomato and lettuce, as well as the germination of weed species such as large crabgrass (*Digitaria sanguinalis*). Moreover, allelopathic impacts of *Ambrosia* spp. on the growth and germination of native species in invaded ranges demonstrate that allelopathy plays a potential role in the success of their invasion [11, 12].

*Ambrosia psilostachya* (western ragweed), a member of the Asteraceae, is native to North America and has spread to America, Europe, and Asia [13]. The occurrence of the *A. psilostachya* in Iran was first reported in 2017 [14]. It is a noxious weed spread that can occur by seeds and rhizomes. Allelopathy effects due to the volatile materials of different parts of *A. psilostachya* have previously been reported by [15], but their attempts to identify its chemical components were unsuccessful. Although there are reports on identifying compounds from different species of Ambrosia, little knowledge has been reported about the chemical composition of *A. psilostachia*. *A. psilostachya* often colonizes coastal areas, dunes, sandy soils, rivers, and ruderal habitats [16], due to the high spread potential, it is reported in some south strip areas of the Caspian Sea (Guilan), Iran, where it has been considered an invasive weed in urban areas and fields [14, 17]. Considering the vast invasion of this species in the world, this study aims to: (i) Identification of the chemical compositions of the essential oil extracted from *A. psilostachya*, and (ii) evaluate the potential allelopathic effects of *A. psilostachya* on crop plants was conducted. *Phaseolus vulgaris* L. is one of the most important legumes in the world, playing an important role in human nutrition, and *Arachis hypogaea* L., an annual legume, is a rich source of multivitamins, thiamine, folic acid, and tocopherols [18].

## 2 Materials and Methods

### 2.1 Collection and Sample Preparation

The study was conducted during 2020‐2021 in the laboratory and greenhouse of the University of Guilan and the University of Zanjan, Iran. Plant samples consisting of mature plants of *A. psilostachya*, at the vegetative stage, were harvested from infected fields in Guilan Province, Bandar Anzali, Iran, in August 2020 (36.6939°N, 48.4099°E). The plant was identified taxonomically by Dr.Somayeh Tokasi, assistant professor of weed Science (Plant Protection Research Department, Gilan Agricultural and Natural Resources Research and Education Center, Agricultural Research, Education and Extension Organization, AREEO, Rasht, Iran). A specimen was kept at Halophytes and C4 Research Laboratory, School of Biology, University of Tehran, Tehran. Fresh *A. psilostachya* plants were separated into leaves, shoots, and roots, and were used for essential oil extraction. To prepare the aqueous extract, part of the samples was diced into 1 cm long pieces and dried in the oven at 70°C for 48 h. Extracts were made by placing 100 g of dried material separately into 1000 mL of distilled water and leaving it at room temperature for 72 h.

### 2.2 Identification of Chemical Compounds of *A. psilostachya*

#### 2.2.1 Essential Oil Isolation

Plant shoots and roots from air‐dried plant material were used separately for extraction. For each sample, the plant material (100 g) was placed in a 2000 mL distillation flask and 1.000 mL of distilled water was added [19]. The essential oils of each sample were obtained by water distillation using a Clevenger apparatus (Ildam, Ankara, Turkey) according to the European Pharmacopoeia method [20]. The oils were dried overusing anhydrous sodium sulfate until analysis. The oil yield was calculated according to the dry weight of plant materials and the amount of essential oils obtained.

#### 2.2.2 Gas Chromatography‐Mass Spectrometry Analysis

Chemical analysis of the essential oil of *A. psilostachya* was obtained with gas chromatography‐mass spectrometry (GC‐MS) and Mass Hunter workstation software. Each sample was subjected to GC‐MS using a flame ionization detector (FID), and the column was a fused silica capillary DB‐5MS (5% phenylmethylpolysyloxane, 30 m × 0.25 mm; film thickness 0.25 µm; J&W Scientific Fisons, Folsom, CA). The temperature program used was as follows: 5 min at 60 then 4°C min−1 up to 220°C, then 11°C min−1 up to 280°C, held for 15 min. Helium was the carrier gas at a flow rate of 1 mL/min. The flow ratio was equal to 1/50. MS in the mass of 70 eV and 30–550 amu, and the ion source's temperature was set to 230°C. According to the compounds known in the literature, their retention indices are compared by contrasting the same series of n‐alkanes on the HP‐5MS capillary column and stored in the spectrograph database by comparing their mass spectra with NIST05.LIB and NIST05s.LIB, as well as by CO injection of available reference compounds.

### 2.3 Phytotoxic Effect of the Aqueous Extracts of *A. psilostachya*

#### 2.3.1 Preparation of Aqueous Extracts

The second experiment was to determine the phytotoxic activities of *A. psilostachya* extract on bean and peanut seedlings. For the preparation of the aqueous extract, the dried plant material was powdered by an electric blender. A stock solution was prepared by adding ground powder into distilled water and shaking for 48 h on a shaker. Finally, from a stock solution, four concentrations were then made.

### 2.4 Greenhouse Experiments

A greenhouse experiment was conducted from May through October 2021. The experiment was conducted in a completely randomized design, consisting of different concentrations of *A. psilostachya* extracts (0%, C0; 25%, C1; 50%, C2; 75% C3; 100% C4), with four replicates. In this experiment, seeds of bean and peanut were sterilized using Sodium hypochlorite (NaOCl) for 3 min to avoid contamination. Seeds were then washed with distilled water before planting. Experimental pots (18 × 18 × 25 cm) were filled with a sterilized mixture of arable soil. Ten seeds of each plant were sown separately in each pot and thinned after germination. Immediately after seeding the target plants, the pots were watered with different concentrations of eques extracts prepared from *A. psilostachya*. The pots were watered to maintain field capacity. To determine the growth traits, at final harvesting, the leaf area index (LAI), biomass dry weight, lengths of the shoot, and yield were recorded. Shoot height was measured from the soil level to the upper point of the terminal bud in millimeters using a ruler. After the oven drying of the samples at 60°C, yield and dry weight aboveground biomass were measured with an electronic balance (0.001 g accuracy). The total plant leaf area was measured by a digital planimeter, and LAI was calculated by dividing the total plant leaf area (A: dm2) by its ground area (P: dm^2^).

$$\mathrm{LAI}=\frac{A}{P}$$

### 2.5 Statistical Analysis

Data were subjected to statistical analysis, and means were analyzed using the analysis of variance (ANOVA). The differences between the means were determined using the L.S.D. test at a significant level, p < 0.05. Statistical package Unistat (Unistat, Inc., USA) was used.

## 3 Results and Discussion

### 3.1 Identification of Chemical Compounds of *A. psilostachya*

**FIGURE 1:**
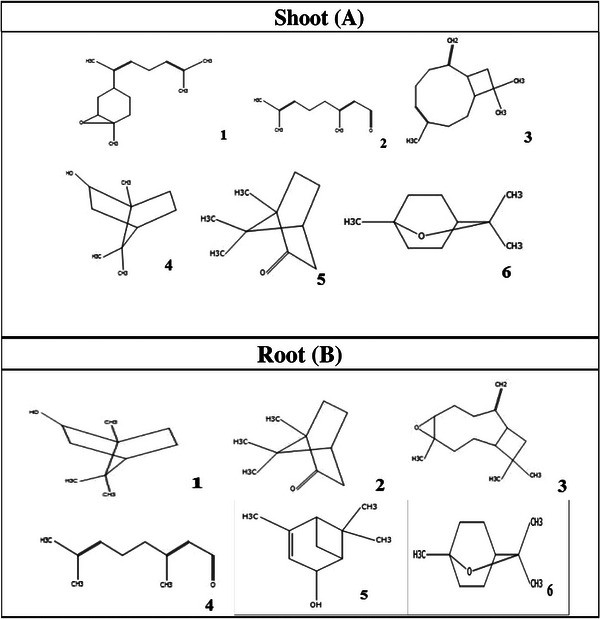
Chemical structure of some major compound identified in the essential oil of shoot (A): 1‐ Isoaromadendrene epoxide, 2‐ Citral, 3‐ Caryophyllene, 4‐ Borneol, 5‐ Bornanone, and 6‐ Eucalyptol, and root (B) of *Ambrosia psilostachya*: 1‐ endo‐Borneol, 2‐ (+)‐2‐Bornanone, 3‐ Caryophyllene oxide, 4‐ Citral, 5‐ *cis*‐Verbenol, and 6‐ Eucalyptol.

GC/MS analysis of the essential oils of *A. psilostachya* allowed the identification of 44 compounds from aerial plant parts and 22 compounds from roots (Table 1). Our results revealed that the main constituents of *A. psilostachya* essential oil from shoot and leaves were isoaromadendrene epoxide (17.38 %), citral (15.13 %), caryophyllene (7.23 %), borneol (8.76%), (+)‐2‐bornanone (6.45 %), eucalyptol (4.61 %), whereas the major compounds from root were endo‐borneol 22.76%, (+)‐2‐bornanone 8.08%, caryophyllene oxide 7.77%, citral 14.65%, *cis*‐verbenol 4.43%, and eucalyptol 4.72% (Table 1). All other components were detected with content below 5%. The chemical structure of major compounds identified in the essential oil of the shoot and root of *A. psilostachya* is presented in Figure 1.

**TABLE 1:** Essential oil composition of *Ambrosia psilostachya*.

Chemical component of the shoot			
Chemical name	Formula	RT	AREA SUM%
Heptane	C7H16	5.42	0.26
Camphene	C10H16	9.48	0.56
Sulcatone	C8H14O	9.94	0.3
Eucalyptol	C10H18O	26.52	4.61
Sabinene hydrate	C10H18O	11.74	0.69
*cis*‐Verbenol	C10H16O	11.91	0.18
Caren‐4‐ol	C10H16O	12.05	0.49
*trans*‐Verbenol	C10H16O	12.428	3.03
(+)‐2‐Bornanone	C10H16O	12.49	6.45
Rosefuran epoxide	C10H14O2	12.71	0.47
Borneol	C10H18O	12.77	8.76
Terpinen‐4‐ol	C10H18O	12.87	1.5
*(‐)‐trans*‐Isopiperitenol	C10H16O	13.15	1.67
Levoverbenone	C10H14O	13.34	1.64
Citral	C10H16O	13.942	15.13
Thymol	C10H14O	14.158	2.47
E‐Methylgeranate	C11H18O2	14.533	0.34
Nerol acetate	C12H20O2	15.176	3.57
Copaene	C15H24	15.398	0.69
Caryophyllene	C15H24	15.958	7.23
β‐Cedrene	C15H24	16.034	0.29
1,4,7,‐Cycloundecatriene, 1,5,9,9‐ tetramethyl‐, Z,Z,Z	C15H24	16.327	0.73
*trans*‐. β‐Ionone	C13H20O	16.511	0.48
β‐cubebene	C15H24	16.6	0.41
Cadinenes	C15H24	16.944	0.33
Kessane	C15H26O	17.141	0.18
Caryophyllene oxide	C15H24O	17.383	0.52
Palustrol	C15H26O	17.561	0.71
Spatulenol	C15H24O	17.631	4
Bisabolene	C15H24O	17.694	2.42
Isoaromadendrene epoxide	C15H24O	17.739	17.38
Viridiflorol	C15H26O	17.809	2.15
Ledol	C15H26O	17.91	0.73
Humulene epoxide 2	C15H24O	17.987	1.99
Benzoic acid	C16H30O4Si3	18.03	0.24
Longipinane	C15H26	18.01	0.49
Isospathulenol	C15H24O	18.17	0.49
7R,8R‐8‐Hydroxy‐4‐isopropylidene‐7methylbicyclo[5.3.1]undec‐1‐ene	C15H24O	18.21	3.85
alpha‐Cadino	C15H26O	18.324	0.58
4(15),5,10(14)‐Germacratrien‐1‐ol	C15H24O	18.37	0.59
Tricyclo[5.2.2.0(1,6)]undecan‐3‐ol, 2methylene‐6,8,8‐trimethyl	C15H24O	18.52	0.27
Longipinocarveol, *trans*‐	C15H24O	18.67	0.72
Ylangenol	C15H24O	19.25	0.18
Hydroxy‐α‐muurolene	C15H24O	19.4	0.22

Chemical component of the root			
Chemical name	Formula	RT	Area Sum %
Methylphosphonothioate	C8H20NO2PS	5.15	0.3
Heptane	C7H16	5.41	4.4
Oxalic acid, butyl propyl ester	C9H16O4	6.84	0.24
Eucalyptol	C10H18O	10.76	4.72
Sabinene hydrate	C10H18O	11.75	0.26
*cis*‐Verbenol	C10H16O	12.42	4.43
(+)‐2‐Bornanone	C10H16O	12.49	8.08
Camphenol, 6‐	C10H16O	12.71	1.9
endo‐Borneol	C10H18O	12.75	22.76
Terpineol	C10H18O	13.03	2.72
*cis‐*Isopiperitenol	C10H16O	13.16	1.87
Levoverbenone	C10H14O	13.34	1.57
Citral	C10H16O	13.92	14.65
Caryophyllene	C15H24	15.95	2.05
Spatulenol	C15H24O	17.63	1.6
Caryophyllene oxide	C15H24O	17.72	7.77
Epiglobulol	C15H26O	17.8	0.65
Humulene epoxide I	C15H24O	17.98	1.17
Torreyol	C15H26O	18.17	0.73
Spathulenol	C15H24O	18.48	1.32
Mintoxide	C15H24O	18.68	0.35
Phthalic acid	C24H34O4	20.08	0.37

The identification of chemical components of *A. psilostachya* essential oil has been documented in some previous studies [21, 22]. Our results showed that there were differences between the main components of the essential oil of *A. psilostachya* and those previously reported in *A. trifida* [23] and *A. artemisiifolia* [24], which belong to the same genus (Table 2). Interestingly, the chemical profile of *A. psilostachya* essential oil differed significantly in the present study, compared to previous reports. Sesquiterpenoids, flavonoids, terpenoids, steroids, and coumarins are the major compounds identified in *Ambrosia* spp. [25]. The main compounds of *A. psilostachya* essential oil obtained in the current study, such as the isoaromadendrene epoxide, citral, *β*‐caryophyllene, bornanone, eucalyptol, differed from the composition reported by [21], rich in germacrene, *trans‐β*‐ocimene, and *β*‐caryophyllene. Furthermore, it can be noted that the major essential oil components of the shoots of *A. psilostachya* were oxygenated sesquiterpenes and monoterpene aldehydes, whereas, in a similar study, the chemical compositions of the essential oils isolated from parts of *A. psilostachya* included sesquiterpene hydrocarbons and monoterpene hydrocarbons as the principal compounds [21]. The results obtained can be attributed to the fact that *Ambrosia* spp. is a very diverse genus of Asteraceae, presenting a high degree of morphological and chemical variability [26]. Different factors, including genetic, biotic, and abiotic factors, could influence the main constituents of essential oil, which may affect the quantity and quality of the plant's secondary metabolites [27]. In fact, plants are forced to change the function and chemical composition of their essential oils to adapt to different environmental conditions [28]. Moreover, moisture and soil can also affect essential oil production and their quality and biological activity [29, 30, 31].

**TABLE 2:** The main composition (relative amount, %) of the essential oils from *Ambrosia psilostachya*, *Ambrosia trifida*, and *Ambrosia artemisiifolia*.

*A. trifida*	*A. artemisiifolia*	*A. psilostachya*
Bornyl acetate (15.5%)	Germacrene D (24.1%)	Epoxide (17.38 %)
Borneol (8.5%)	Limonene (16.8%)	Citral (15.13 %)
Caryophyllene oxide (8.3%)	α‐pinene (8.0%)	Caryophyllene (7.23 %)
α‐Pinene (8.0%)	β‐Myrcene (7.4%)	(+)‐2‐Bornanone (6.45 %)
Germacrene D (6.3%)	Borneol (2.9%)	Borneol (8.76%)

### 3.2 Phytotoxic Activities of Extracts of *A. psilostachya*

**FIGURE 2:**
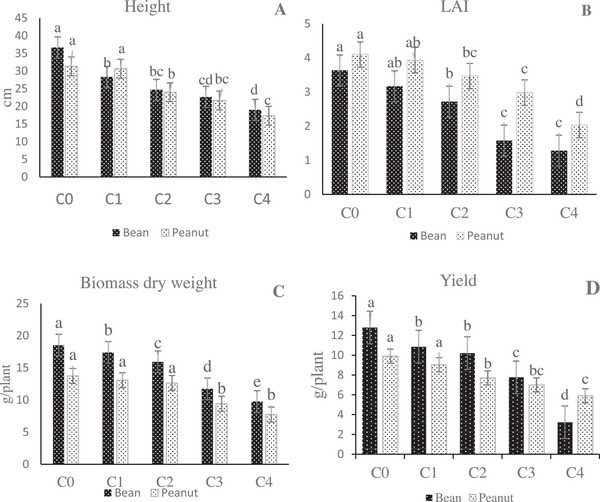
Effects of *Ambrosia psilostachya* aqueous extracts on *Phaseolus vulgaris* and *Arachis hypogaea* height (A), LAI (B), biomass dry weight (C), and yield (D); (0%, C0; 25%, C1; 50%, C2; 75% C3; 100% C4).

ANOVA analysis revealed significant (*p* ≤ 0.05) differences among mean squares of LAI, biomass dry weight, lengths of the shoot, and yield under various concentrations. The influence of *A. psilostachya* aqueous extracts on the target plants is shown in Figure 2A–D. Results showed that increasing the concentrations of *A. psilostachya* extracts caused a reduction in both bean and peanut growth traits.

The application of aqueous extracts of *A. psilostachya* caused a considerable decrease in the height of bean plants at all concentrations. As the concentration of *A. psilostachya* aqueous extracts increased, the height of seedlings gradually decreased compared to the control plants. In peanut plants, there was no significant difference in the height between control plants and 25% concentration, but at higher concentrations of the aqueous extract, the height decreased. LAI showed variations in both crop plants. When compared with control (C0), lower concentrations (i.e., C1 and C2 treatments) of aqueous extracts had no significant effects on the LAI of both the crops, while the highest concentration (C4) inhibited LAI. In bean plants, the magnitude of the percent of decrease at different concentrations followed the order: C1 (12%) < C2 (25%) < C3 (56%) < C4 (64%) in LAI, respectively, compared to control, while in peanut plants, the percent of the decrease in LAI was C1 (4%) < C2 (15%) < C3 (27%) < C4 (50%). The results of biomass dry weight showed that the dry weight of the bean plants was significantly inhibited by all the concentrations of the aqueous extracts. In contrast, the biomass dry weight of the peanut plants was inhibited at a concentration of 75% and above. The aqueous extract of *A. psilostachya* significantly (*p* = 0.05) reduced the yield of crop species. At the concentration of 100%, the yield of beans and peanuts was reduced by nearly 60% and 40%, respectively. Compared to the control plants, the 25% concentration of aqueous extracts of *A. psilostachya* had no significant effect on the yield of peanut plants, but, in bean plants, this parameter showed a significant reduction at all concentrations.

Allelopathic activity of *Ambrosia* spp. has been documented on germination, seedling growth, and yield of crop species such as corn, maize, rice, wheat, and soybean [9, 15, 32], and extracts from different species of the genus *Ambrosia* exhibited significant inhibitory effects on other plants, especially crop seedlings [7, 33]. In conformity with previous studies, the results of this study showed that the presence of *A. psilostachya* strongly affected the growth traits of test crop plants with a dose‐dependent trend, so that the increase in the concentration of *A. psilostachya* extract decreased growth traits of both bean and peanut plants. In previous literature, it was found that the presence of allelochemical compounds inhibited cytokine activity and decreased the height of plants [34, 35], and, consistent with these studies, the height of both crop plants decreased with increasing the concentration of *A. psilostachya* extract. Results showed that LAI was also inhibited by *A. psilostachya* aqueous extract in the tested plants after treatment. Under stress conditions, due to a decrease in cell number and cell size, leaf area decreases [36]. The high concentrations of *A. psilostachya* extracts, by inhibition of LAI, led to a reduction in the growth of the tested plants. This reduction may be related to the inhibition of cell division and/or cell expansion due to the presence of chemical components in the extracts [37, 38]. Furthermore, the allelopathic compounds can affect enzymatic activity, chlorophyll synthesis, cell viability, and organelle size [39, 40]. These results suggest that the invasion of *A. psilostachya* in agricultural areas could severely affect crop production. In this study, the bean was more sensitive than the peanut to *A. psilostachya* aqueous extract, so that the yield of peanut plants was only reduced by 40% at the 100% concentration, while the yield of bean was reduced by 74% at the same concentration. Moreover, the result showed that the extract did not significantly inhibit the yield of peanut plants at low concentrations, which could suggest that the allelopathic compounds in *A. psilostachya* have a limited effect on this parameter in peanut. In other words, peanut plants were less affected by *A. psilostachya* extract concentration than beans. These findings are in agreement with the results of another study, which reported that different crops showed different sensitivity to the *A. tirifida* [7]. These results show that the response of plants to allelopathic interference of invasive species may differ from species to species. Under stressful situations presence of enzymes such as catalase, superoxide dismutase, and peroxidase regulates ROS accumulation and reduces cellular damage [41]. Therefore, plants show different tolerance to adverse conditions.

The observed inhibitory effects of *A. psilostachya* extracts could be attributed to the presence of allelochemical compounds, which have phytotoxic activity. Allelochemicals can inhibit the process of photosynthesis so that the plant growth and the biomass dry weight of the seedlings decrease [42]. The presence of stress in the form of allelochemical compounds could disrupt cell division and enlargement, thus inhibiting the growth of plants. The mode of action of many allelochemicals is the production of reactive oxygen species (ROS) and induction of oxidative stress [43]. The composition and major components of plant extracts play a significant role in their biological activity. Maximum inhibition of aqueous extracts of *A. psilostachya* could be due to the release of phytotoxins in the soil [44]. In this regard, many reports confirmed that *Ambrosia* spp. exhibited powerful phytotoxic effects on germination and seedling growth, such as lettuce, watermelon, corn, and tomato [9, 45, 46]. In line with a previous study, the extract of *A. artemisiifolia* decreased the dried weight of lettuce plants [10]. It was also reported that the aerial parts of *A. trifida* have the potential to produce harmful phytochemicals, which have an inhibitory effect on plant growth [23]. In the present study, the potent phytotoxic activity of the *A. psilostachya* could be ascribed to the presence of oxygenated compounds such as isoaromadendrene epoxide already reported in other plant species, including *Artemisia* spp. [47], *Rosmarinus officinalis* [48], and *Cinnamomum camphora* [49, 50]. Reported that the antimicrobial activity of the plant components in part may be associated with the high percentage of isoaromadendrene epoxide, caryophyllene oxide, eudesmol, and aromadendrene epoxide. Therefore, due to the high concentration of isoaromadendrene epoxide, citral, caryophyllene, and caryophyllene oxide in this plant, the allelopathy activity of *A. psilostachya* could be associated with their presence.

## 4 Conclusions and Perspectives

To the best of our knowledge, this is the first report identifying the chemical components of *A. psilostachya* essential oil in Iran. The results of this study confirm that *A. psilostachya* has a strong phytotoxic potential on crop plants such as bean and peanut, so that its adverse effects increase with increased concentration. Based on the high concentration of isoaromadendrene epoxide, citral, caryophyllene, and caryophyllene oxide in essential oil and their allelopathic activity, the allelopathy of *A. psilostachya* essential oil could be associated with their presence. Due to the different sensitivity of crop plants to *A. psilostachya* essential oil, the results of this study could help choose crop plants when heavy *A. psilostachya* infestations occur. Finally, it should be noted that the results of this study could also promote the use of *A. psilostachya* as a source to manage weeds.

## Conflicts of Interest

The authors declare no conflicts of interest.

## Data Availability

The data that support the findings of this study are available from the corresponding author upon reasonable request.
